# Choroidal Neovascularization in a Pediatric Patient With Optic Disc Pit

**DOI:** 10.7759/cureus.77231

**Published:** 2025-01-10

**Authors:** Marwa Mukhtar, Malik Mudasser Yasin, Mohib Naseer

**Affiliations:** 1 Ophthalmology, Medical Teaching Institution (MTI) Ayub Teaching Hospital, Abbottabad, PAK; 2 Ophthalmology, Medical Teaching Institution (MTI) Khyber Teaching Hospital, Peshawar, PAK; 3 Ophthalmology, University Hospital Limerick, Limerick, IRL; 4 Ophthalmology, Cork University Hospital, Cork, IRL

**Keywords:** choroidal neovascularization, fundus fluorescein angiography (ffa), intravitreal anti-vegf, optical coherence tomography (oct), optic disc pit

## Abstract

Optic disc pit is a congenital anomaly of the optic disc which is usually asymptomatic, but some patients can develop optic disc pit maculopathy at some stage of life.

Choroidal neovascularization (CNV) is an abnormal growth of blood vessels into the neurosensory retina from choroidal vessels. Although CNV is not commonly seen in the pediatric age group, there are certain optic nerve head anomalies, like optic disc pit, optic nerve drusen, optic nerve head cavity abnormalities, etc., which are associated with CNV in children.

Here, we present a 13-year-old girl who was diagnosed with CNV in association with optic disc pit. She was given intravitreal anti-vascular endothelial growth factor (anti-VEGF) injections. CNV got improved after anti-VEGF injections, along with significant improvement in vision.

## Introduction

Optic disc pit is a congenital anomaly of the optic disc which appears as an oval, grey-white excavation at the optic disc and is most commonly present at the inferotemporal part of the disc. Although optic disc pit is generally asymptomatic, approximately 25-75% of patients can develop optic disc pit maculopathy, i.e., retinoschisis and/or serous retinal detachment, at some stage of life [1].

Choroidal neovascularization (CNV) is the formation of new blood vessels into the neurosensory retina from choroidal vessels through Bruch's membrane [2]. Some of the causes of CNV in the pediatric age group are trauma, high myopia, posterior uveitis, angioid streaks [3], optic nerve head cavity abnormalities, etc. [4].

## Case presentation

**Figure 1:**
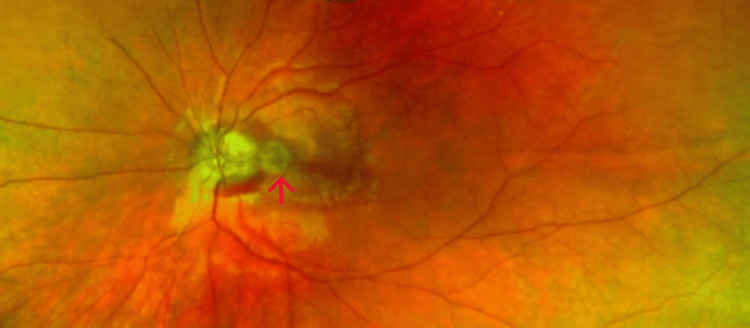
Fundus photograph of the left eye showing the optic disc pit and peripapillary hemorrhage

A 13-year-old girl presented to eye casualty with a complaint of gradual, painless loss of vision in her left eye for six weeks. Her past ocular and medical history was not significant. On examination, her best corrected visual acuity in the right eye was 6/6 and in the left eye was 6/60. Intraocular pressure was within normal range in both eyes. There was no relative afferent pupillary defect. Anterior segment examination was normal in both eyes. Posterior segment examination showed bilateral irregular optic disc margins along with optic disc pit and peripapillary hemorrhage in the left eye (Figure 1).

**Figure 2:**
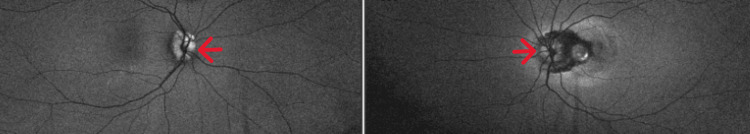
Fundus autofluorescence showing the hyper-autofluorescence at the optic nerve head bilaterally

**Figure 3:**
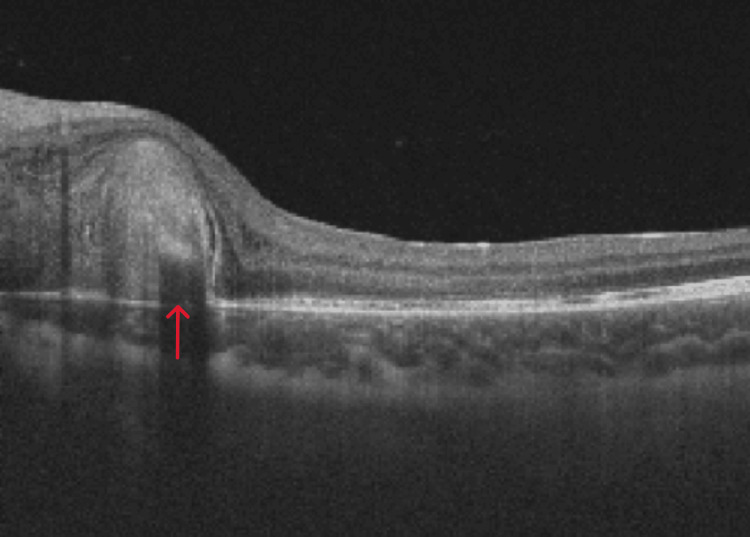
OCT at presentation showing the sub-retinal fluid and sub-retinal hyperreflectivity OCT: optical coherence tomography

**Figure 4:**
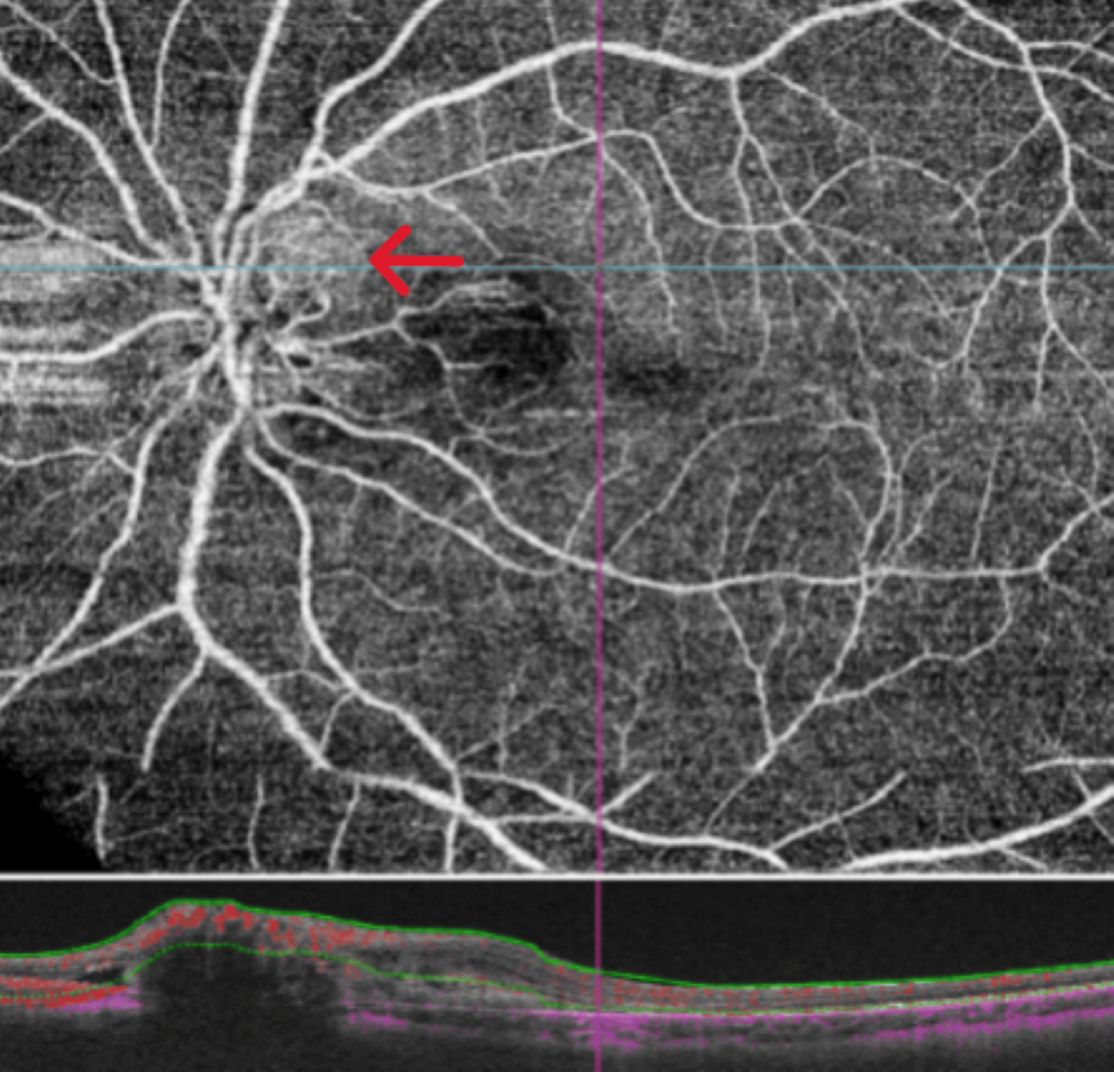
OCT angiography showing the sub-retinal vascular net in the peripapillary area OCT: optical coherence tomography

**Figure 5:**
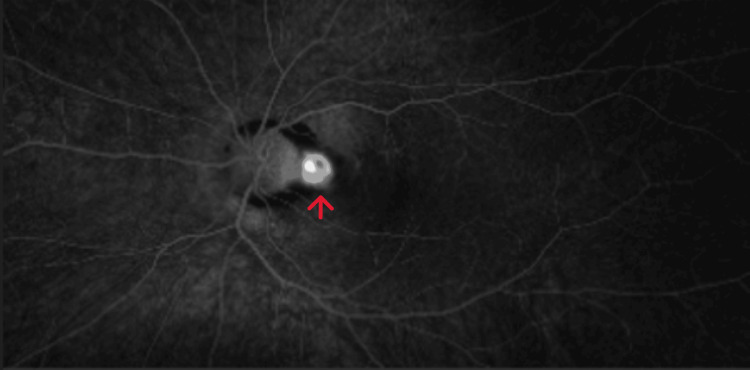
FFA showing the hyperfluorescence in the peripapillary area FFA: fundus fluorescein angiography

Fundus autofluorescence was done which confirmed bilateral optic nerve head drusen (Figure 2). Optical coherence tomography (OCT) of the macula of the right eye was normal but showed peripapillary sub-retinal fluid in the left eye (Figure 3). OCT angiography (OCT-A) showed a sub-retinal vascular net in the peripapillary area of the left eye (Figure 4). Fundus fluorescein angiography (FFA) was performed which confirmed leakage consistent with OCT-A (Figure 5). Diagnosis of left optic disc pit with peripapillary choroidal neovascular membrane (CNVM) was formed.

**Figure 6:**
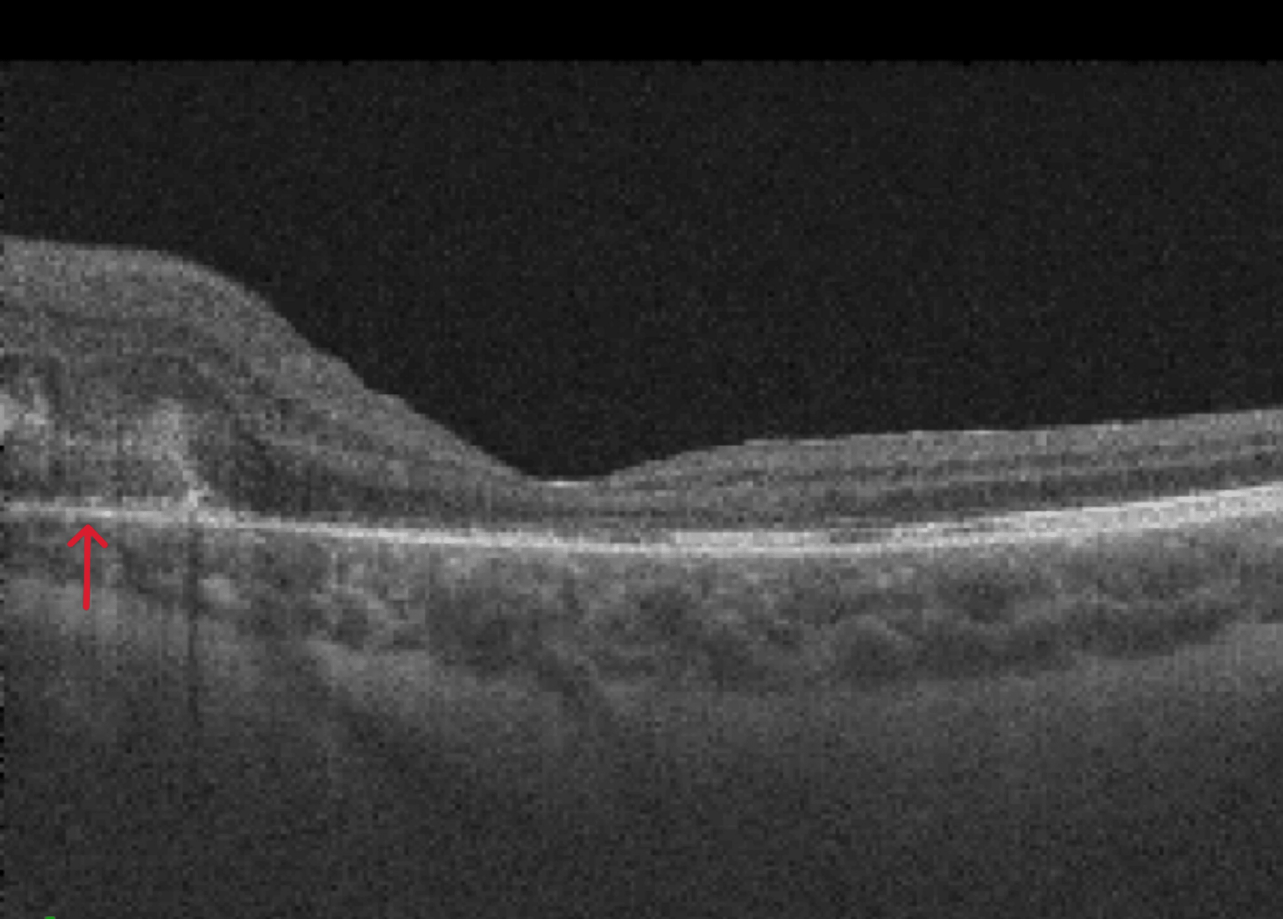
OCT one-week post-anti-VEGF injection showing the significant improvement in sub-retinal fluid OCT: optical coherence tomography; anti-VEGF: anti-vascular endothelial growth factor

She was given the first dose of intravitreal Avastin injection and then a review in the outpatient clinic in one-week time was scheduled. Her visual acuity improved from 6/60 to 6/9 and sub-retinal fluid was significantly improved (Figure 6). She was booked for a second intravitreal Avastin injection. On a follow-up visit after the second intravitreal Avastin injection, her visual acuity improved to 6/7.5 and sub-retinal fluid was resolved on OCT of the macula. It was planned to monitor her closely, and she was booked for a further follow-up appointment in four-week time.

## Discussion

Optic disc pit is a congenital anomaly of the optic disc that appears as a round or oval depression, located most commonly at the inferotemporal part of the optic disc [1]. It affects approximately one in 11,000 people. Although optic disc pit generally remains asymptomatic, around 25-75% of people develop optic disc pit maculopathy, i.e., retinoschisis and/or serous retinal detachment, usually in the third or fourth decade of life [5].

CNV is an abnormal growth of blood vessels into the neurosensory retina from choroidal vasculature through Bruch's membrane [2]. It is one of the most common causes of vision loss in developed countries [6]. Some of the common causes of CNV in the adult population are age-related macular degeneration, pathologic myopia, posterior uveitis, retinal dystrophies, etc. [2].

CNV in the pediatric age group is uncommon. Causes of pediatric CNVM include trauma, high myopia, posterior uveitis [3], optic disc drusen, chronic papilledema, optic nerve head cavity abnormalities, idiopathic anomalies, etc. [4].

Compared to adults, CNV in the pediatric age group has a better natural course, and spontaneous regression has been reported in approximately 58% of cases [4]. Despite this possibility of spontaneous involution of CNV in children, research has proven that anti-vascular endothelial growth factor (anti-VEGF) confers a significant advantage over spontaneous involution in terms of visual outcome. This underscores the importance of prompt diagnosis and therapeutic intervention in achieving optimal visual results [7].

Research has proven the effectiveness of intravitreal anti-VEGF therapy as a low-risk treatment for pediatric CNV, with 60% of cases requiring only one injection to stabilize disease progression [7]. This might be due to the better function of retinal pigment epithelium (RPE) pump action in children [4]. Therefore, anti-VEGF is the preferred treatment modality for pediatric CNV as compared to other more destructive treatment modalities. However, long-term follow-up studies may be needed to fully understand the recurrence rates and potential side effects in this population [7].

A case has been reported where an eight-year-old girl was found to have a unilateral optic disc pit and CNVM in the same eye as a cause of reduced vision, which improved subsequently after the administration of a single dose of intravitreal aflibercept injection. She was reviewed in an outpatient clinic for 15 months. The OCT and OCT-A demonstrated stable findings, consistent with earlier results. The patient required no further intervention, and her visual acuity remained stable throughout the follow-up period [7].

Regarding our patient, she also had optic disc drusen, which can be another cause of CNV in the pediatric age group [4]. She received two doses of bevacizumab injection, which resulted in improved vision (from 6/60 to 6/7.5) and the resolution of sub-retinal fluid. Currently, she is being monitored in the outpatient clinic on a regular basis.

## Conclusions

In this case report, an association between optic disc pit, optic disc drusen, and CNV in a child has been shown. CNV got significantly improved after intravitreal anti-VEGF injections along with significant improvement in visual acuity. This emphasizes the importance of thorough clinical examination to look for disc anomalies in patients presenting with CNV. Moreover, early intervention can lead to better visual outcomes.
